# Research on a new type of ureteral stent material Zn-2Cu-0.5Fe-xMn with controllable degradation rate

**DOI:** 10.1016/j.heliyon.2024.e37629

**Published:** 2024-09-07

**Authors:** Jiawen Zheng, Yichun Zheng, Peng Sun, Desheng Zhu, Wentao Fan, Ting Huang, Yanfei Fang, Qing Yang, Min Xu

**Affiliations:** a Department of Urology, Affiliated Jinhua Hospital of Zhejiang University School of Medicine, Jinhua, 321000, China; b Zhejiang University School of Medicine, Hangzhou, 310009, China

**Keywords:** Zn alloy, Ureteral stent, High performance, Degradation rate

## Abstract

The placement of ureteral stents plays a crucial role in the treatment of ureteral strictures, therefore requiring high material performance standards. In addition, depending on the etiology of ureteral strictures, there are significant differences in the retention time of ureteral stents, thus requiring different degradation rates for the stents. Therefore, it is crucial to develop stent materials with high performance and controllable degradation rates. This study explores the potential of Zn-2Cu-0.5Fe-xMn alloy as a ureteral stent material, utilizing the antibacterial effect of copper ions, the strengthening effect of Fe element on Zn-based alloys, and the accelerated degradation effect of Mn element. The research found that with the increase in Mn content, the average grain size of the alloy and the size of (Fe, Mn)Zn13 phase gradually increased, leading to a decrease in hardness. The corrosion rate of the alloy increased with the increase in Mn content, attributed to changes in grain size and standard electrode potential differences between elements. Due to the antibacterial effects of Zn ions and Cu ions, the Zn-2Cu-0.5Fe-xMn alloy exhibits good anti-stone formation capabilities. Furthermore, the alloy also demonstrates acceptable cytotoxicity. Therefore, the Zn-2Cu-0.5Fe-xMn alloy is expected to become an important implant material in urological surgery.

## 1 Introduction

Ureteral stricture is a common disease in the human urinary system, often caused by inflammation, stones, trauma, tumor compression or invasion, iatrogenic injury, among other reasons. It can lead to obstruction of the upper urinary tract, and in severe cases, even result in chronic renal failure. Ureteral stent placement is an important treatment method for relieving upper urinary tract obstruction [[1], [2], [3], [4]]. The purpose of ureteral stent placement is to adequately drain the upper urinary tract while minimizing hospitalization time and negative impacts on quality of life. Currently, there is a variety of materials used for ureteral stents, such as silicone, polyurethane, titanium alloy, stainless steel, and CoCr alloy, which exhibit high corrosion resistance and acceptable cytotoxicity [[5], [6], [7], [8], [9]].

Ureteral infections and secondary stone formation caused by implants are two common complications of ureteral stent placement [[10], [11], [12]]. Bacteria in the urine can colonize on the implant and form a biofilm, leading to long-term chronic infections; additionally, bacteria can promote urea hydrolysis, alkalize the urine, accelerate the precipitation of inorganic salts such as calcium and magnesium in the urine, and promote stone formation [12]. However, antibiotic therapy is not very effective against infections caused by bacterial biofilms [13,14]. When stent-related complications occur or when the course of treatment with the ureteral stent ends, the stent must be removed through a secondary surgery. Therefore, stent materials with good antibacterial capabilities, mechanical properties, acceptable cytotoxicity, and degradability are receiving increasing attention.

Current research on biodegradable stent materials includes both metal and polymer materials, with metal stents having superior mechanical strength compared to polymer stents [[5], [6], [7], [8], [9]]. Magnesium, iron, and zinc-based alloys are three widely reported metals used for biodegradable stents [[15], [16], [17], [18]]. Magnesium-based alloys exhibit acceptable cytotoxicity, anti-inflammatory responses, and mechanical properties. However, magnesium-based alloys degrade too quickly in the body, losing mechanical support within 2–3 months after stent implantation and completely degrading within 4 months [19]. Additionally, degradation of magnesium-based alloys in the body produces hydrogen gas [19]. While iron-based stents have excellent mechanical properties, the degradation rate of iron-based alloys is too slow, and the degradation products are not easily absorbed by surrounding tissues [15]. Compared to magnesium-based and iron-based alloys, zinc-based alloys exhibit more suitable degradation rates. Furthermore, zinc is one of the essential trace elements in the human body, participating in and influencing the activity of 300 enzymes, as well as being involved in processes such as signal transduction, cell apoptosis, nucleic acid metabolism, and gene expression regulation [[20], [21], [22]]. Currently, multiple research teams, including those from Michigan Technological University, have found that zinc-based alloys are highly effective as biodegradable scaffold materials, and zinc-based alloys have become the primary candidate materials for the next generation of biodegradable stents [23,24].

Different medical conditions require different degradation rates of ureteral stents. For example, post-lithotripsy stents may need to be in place for 8–12 weeks, while malignant tumor invasion may require at least 1 year or more [25,26]. However, there is currently limited research on controllable degradation rates of zinc-based alloys. In response to this issue, this study utilized the degradability of zinc, the antibacterial properties of copper ions, the reinforcement effect of iron elements on zinc-based alloys, and the accelerated degradation effect of manganese elements, the novel Zn-2Cu-0.5Fe-xMn alloy was developed with long-lasting antibacterial effects, acceptable cytotoxicity, and anti-stone capabilities, while also allowing for the control of degradation rates through composition design. This work provides a scientific basis and innovative approach for the development of novel biodegradable zinc-based materials for treating ureteral infections.

## 2 Experimental methods

### 2.1 Fabrication of alloys

The Zn, Cu-35Zn, Zn-10Fe and Zn-20Mn with purity above 99.995 % were proportionally placed into a vacuum levitation melting furnace and melted at 550 °C. To ensure uniform distribution of the alloy, the melting process was repeated three times. The molten alloy was then poured into preheated (250 °C) steel molds to obtain metal rods with a diameter of 15 mm and a length of 100 mm. Subsequently, the alloy underwent a 10 h homogenization heat treatment at 380 °C, followed by furnace cooling to room temperature, resulting in the formation of Zn-2Cu-0.5Fe, Zn-2Cu-0.5Fe-0.1Mn, and Zn-2Cu-0.5Fe-0.3Mn samples. The samples were then cut into circular discs with a diameter of 15 mm and a thickness of 2 mm using electrical discharge machining, followed by grinding with 2000-grit SiC paper for microstructure, electrochemical, and immersion testing. Samples prepared under the same surface conditions were subjected to hardness testing, cytotoxicity assessment, and antimicrobial assessment.

### 2.2 Microstructure characterization and hardness test

The phase composition of the alloy was analyzed using an X-ray diffractometer (XRD, Rigaku Ultima IV). XRD patterns were obtained in the 2θ range of 5°–90° at a scan rate of 4° min^−1^. The microstructure and phase composition of the alloy were examined using an optical microscope (OM, DMI 8) and a scanning electron microscope (SEM, ZEISS Gemini 300). Grain structure analysis was performed through electron back-scattered diffraction (EBSD) using a JEOL JSM-7800 F SEM outfitted with an EBSD system (OXFORD NordlysMax), employing a step size of 0.5 μm. The micro-hardness of the alloy was measured using a Vickers microhardness tester (HVS-1000A, Whdes).

### 2.3 Electrochemical corrosion testing

The alloy was subjected to electrochemical corrosion testing in artificial urine using an electrochemical workstation (CHI660E) at a temperature of 37.0 ± 0.5 °C, constant temperature is achieved by placing the beaker in the electro thermostatic water bath. A typical three-electrode cell system was employed for the electrochemical tests, with the circular disc samples of exposed area 0.2 cm^2^, a saturated calomel electrode (SCE), and a platinum electrode used as the working electrode, reference electrode, and counter electrode, respectively, after polishing. The potentiodynamic polarization curves of the samples were measured at a scan rate of 1 mV/s.

### 2.4 Immersion test

Each material was placed in a 10 mL test tube and suspended in artificial urine to ensure the samples were completely immersed. The artificial urine was changed daily to simulate urinary conditions, and the samples were kept in a 37 °C incubator. After 30 days, the samples were removed and rinsed consecutively with deionized water and alcohol. The surface morphology of the immersed samples was observed using SEM, and compared with pure iron under the same conditions.

### 2.5 Antibacterial evaluation

The antibacterial activity of the samples was evaluated using the colony-forming unit (CFU) counting technique specified in EN ISO 6888-1-2021 [27]. The round disc samples were sterilized at high temperature and pressure (121 °C, 15 min) before CFU detection. Two 12 mL bacterial culture tubes were taken, each containing 3 mL of LB liquid culture medium. Single colonies of *Escherichia coli*/*Staphylococcus aureus* were picked from solid culture media and added to the liquid culture medium in one tube, while the other tube served as a blank control. The tubes were incubated overnight in a constant temperature shaker (37 °C, 200 rpm) for 15 h. The samples were transferred to a 96-well plate, and the bacterial solution was diluted to 10^6^ CFU/mL using sterile phosphate buffered saline (PBS) solution. 50 μL of the bacterial solution was added to each well containing the sample, while the control group did not have any samples added. The plate was then incubated at 37 °C for 24 h. After incubation, the co-culture fluid was washed with 150 μL of sterile PBS solution, and the wash solution was aspirated. The wash solution was diluted 100 times with sterile PBS solution, and 100 μL of the diluted solution was evenly spread on LB solid culture medium. The medium was then incubated in a constant temperature incubator at 37 °C for 18 h, after which the colonies were counted and photographed.

### 2.6 Cytotoxicity evaluation

The in vitro cytotoxicity was evaluated using human kidney epithelial 293T cell line (iCell-h237, Shanghai) according to ISO 10993–5: 2009. The 293T cells were maintained in high-glucose DMEM medium with 10 % fetal bovine serum at 37 °C in a humidified atmosphere containing 5 % CO_2_. For extract preparation, the samples were steam sterilized at 121 °C for 20 min, then immersed in culture medium following ISO 10993–12: 2009 guidelines at a ratio of 1.25 cm^2^/mL for 24 h to collect the leachate from the alloy material.

Subsequently, the 293T cells were seeded in a 96-well plate at a density of 6 × 10³ cells per well and incubated under the same conditions for one day. The original culture medium was then replaced with metal extracts at concentrations of 100 %, 50 %, and 25 %. The cells were further incubated under the same conditions for an additional three days. The culture medium was aspirated, with the control group cultured in medium without the extract. To evaluate the cell viability of the samples, 1 mL of PBS containing 10 % CCK-8 reagent was added to each well and incubated in the incubator for an additional 2 h. Finally, 1 mL of the solution from each well was transferred to a new 96-well plate, and the absorbance at 450 nm was detected using a microplate reader. Three replicate wells were set up for each sample, and the average and standard deviation were calculated to determine the cell viability rate.

To evaluate cell morphology, fluorescence live/dead staining and DAPI staining were performed. Log-phase 293T cells were harvested, counted, and adjusted to the desired cell concentration. Approximately 4 × 10^4^ cells were seeded in a confocal dish and incubated overnight in a constant temperature CO_2_ incubator at 37 °C in a humidified atmosphere containing 5 % CO_2_. After pre-incubation, replace the original cell culture medium with 300 μL of the extract at a 25 % concentration and continue culturing under the same conditions for 3 days. Perform live/dead staining and DAPI staining, and observe cell morphology using a fluorescence microscope (Axio Observer A1, Zeiss, Germany) at 490 nm.

## 3 Results and discussion

### 3.1 Microstructure

**Fig. 1:**
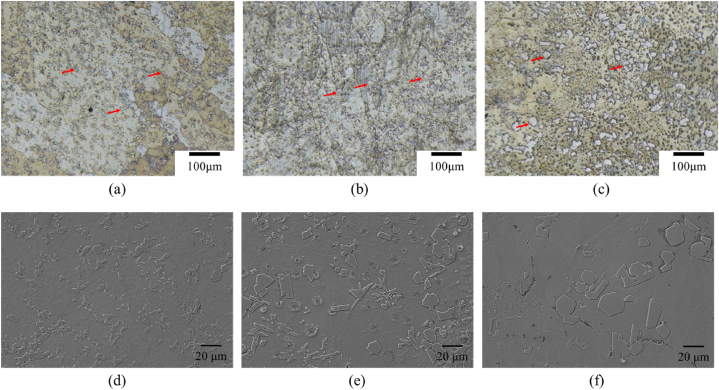
OM and SEM images of Zn-2Cu-0.5Fe (a, d), Zn-2Cu-0.5Fe-0.1Mn (b, e) and Zn-2Cu-0.5Fe-0.3Mn (c, f).

**Fig. 2:**
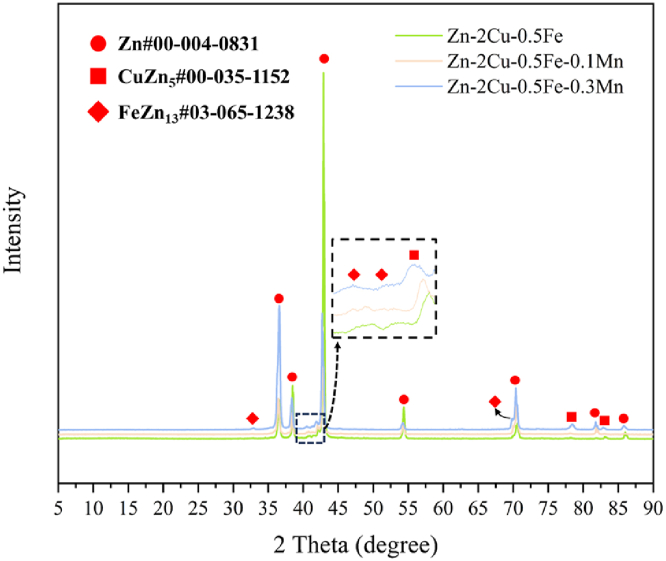
XRD pattern of Zn-2Cu-0.5Fe, Zn-2Cu-0.5Fe-0.1Mn and Zn-2Cu-0.5Fe-0.3Mn.

**Fig. 3:**
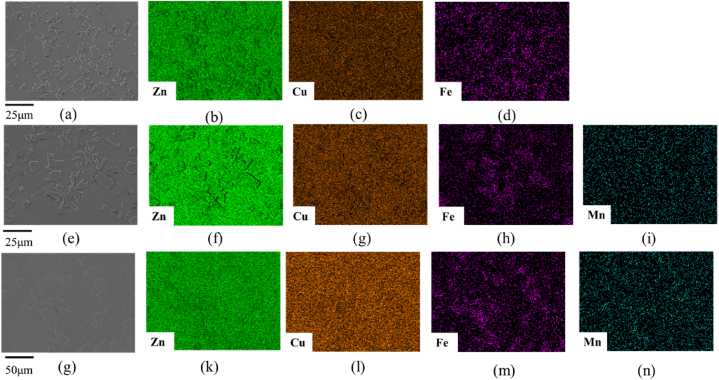
SEM-EDS images of Zn-2Cu-0.5Fe (a–d), Zn-2Cu-0.5Fe-0.1Mn (e–i) and Zn-2Cu-0.5Fe-0.3Mn (g–n).

The OM and SEM images of Zn-2Cu-0.5Fe-xMn alloys with different Mn contents are shown in Fig. 1(a–f). The microstructures of these alloys are similar, with irregular second phases precipitated on the matrix, and the size of the second phase increases with increasing Mn content. To clarify the phase composition of the alloys, XRD and SEM-Energy Dispersive Spectrometer (SEM-EDS) analyses were conducted on the three alloys, and the results are shown in Fig. 2, Fig. 3. The XRD results show that the alloys contain three main phases: Zn (#00-004-0831), CuZn_5_ (#00-035-1152), and FeZn_13_ (#03-065-1238). Due to the complexity of the alloy composition, which includes multiple phases with Zn as the predominant component, it is not feasible to fully display the peaks for CuZn_5_ and FeZn_13_. EDS results show that the Zn element is distributed relatively uniformly throughout the system, while the Fe element exhibits segregation. Regions with Fe segregation have a lower concentration of Cu, and these regions are primarily FeZn_13_. In contrast, areas with a higher concentration of Cu are predominantly CuZn_5_. Compared to Fe, the distribution range of Cu is relatively broader, thus the matrix phase in all three alloys mainly consists of Zn and CuZn_5_. For Zn-2Cu-0.5Fe, the precipitated second phase is FeZn_13_. For Zn-2Cu-0.5Fe-0.1Mn and Zn-2Cu-0.5Fe-0.3Mn, the precipitated second phase is (Fe, Mn)Zn13. Since Fe and Mn both have a body-centered cubic (BCC) structure with only a 3 % difference in atomic radius (156 p.m. and 161 p.m., respectively), Mn atoms can substitute for Fe atoms in forming phases, ultimately leading to an increase in the size of the precipitated phase with increasing Mn content, as shown in Fig. 3(a–e, g).

**Fig. 4:**
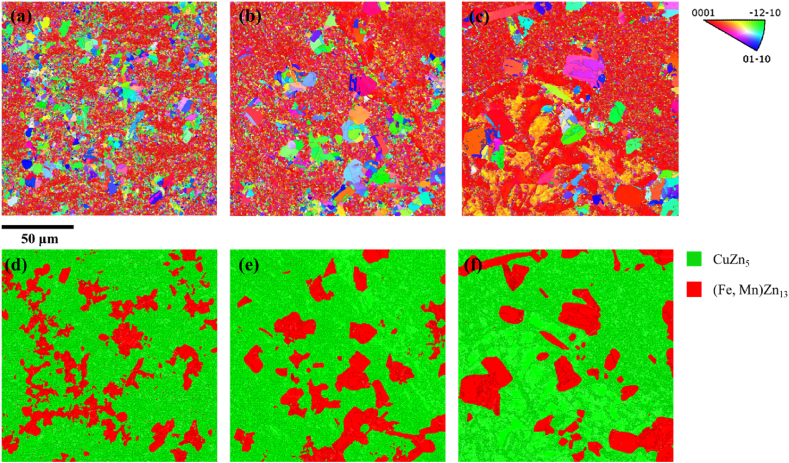
EBSD image of orientation and phase distribution: (a, d) Zn-2Cu-0.5Fe; (b, e) Zn-2Cu-0.5Fe-0.1Mn; (c, f) Zn-2Cu-0.5Fe-0.3Mn.

Fig. 4(a–f) shows the results of EBSD for the three samples, while Table 1 presents the analysis of grain sizes for these samples. In all three samples, the crystal orientations of the CuZn_5_ phase are primarily oriented along <0001>, while the crystal orientations of FeZn_13_ or (Fe, Mn)Zn13 are predominantly oriented along <-12-10> with some grains showing partial <01–10> orientations. For the Zn-2Cu-0.5Fe alloy, the average grain size is 1.2 μm, where the grain size of the FeZn_13_ phase is significantly larger than that of the CuZn_5_ phase, with the largest grain size of FeZn_13_ reaching 9.7 μm. The grain size of CuZn_5_ is relatively uniform, with most grains below 1.0 μm. As for Zn-2Cu-0.5Fe-0.1Mn, the average grain size is 1.3 μm, showing that the FeZn_13_ phase still has a notably larger grain size compared to CuZn_5_. Additionally, compared to Zn-2Cu-0.5Fe, the grain size of (Fe, Mn)Zn13 has increased significantly, with the largest grain size of FeZn_13_ reaching 13.6 μm. Most CuZn_5_ grains are below 1 μm, but some regions exhibit larger CuZn_5_ grains, even comparable in size to the (Fe, Mn)Zn13 grains. For Zn-2Cu-0.5Fe-0.3Mn, the average grain size is 1.3 μm, with the largest grain size observed in the (Fe, Mn)Zn13 phase compared to the other two alloys. The largest grain size of FeZn_13_ reaches 21.3 μm, and large CuZn_5_ grains are present in Zn-2Cu-0.5Fe-0.3Mn as well. Mn tends to accumulate at grain boundaries, the formation of large CuZn_5_ grains in Zn-2Cu-0.5Fe-0.1Mn and Zn-2Cu-0.5Fe-0.3Mn may be attributed to the accumulation of Mn elements at grain boundaries, reducing the grain boundary migration rate and leading to the formation of abnormally large CuZn_5_ grains.

**Table 1:** Grain size information.

Sample	Average grain size (μm)	Maximum grain size (μm)
S_1_	1.2	9.7
S_2_	1.3	13.6
S_3_	1.3	21.3

Table 2 presents the hardness values of the three samples, with Zn-2Cu-0.5Fe exhibiting the highest hardness, while the hardness values of Zn-2Cu-0.5Fe-0.1Mn and Zn-2Cu-0.5Fe-0.3Mn are similar. This is because Zn-2Cu-0.5Fe has the smallest grain size, and an increase in the number of grain boundaries can make dislocation movement more difficult, thereby making the material more resistant to deformation. Additionally, fine grains can more effectively disperse strain energy, prevent grain refinement and cracking, further enhancing the hardness.

**Table 2:** Hardness of Zn-2Cu-0.5Fe, Zn-2Cu-0.5Fe-0.1Mn and Zn-2Cu-0.5Fe-0.3Mn.

Sample	Hardness (HV)
Zn-2Cu-0.5Fe	92 ± 3
Zn-2Cu-0.5Fe-0.1Mn	78 ± 3
Zn-2Cu-0.5Fe-0.3Mn	80 ± 2

### 3.2 Degradation behavior

**Fig. 5:**
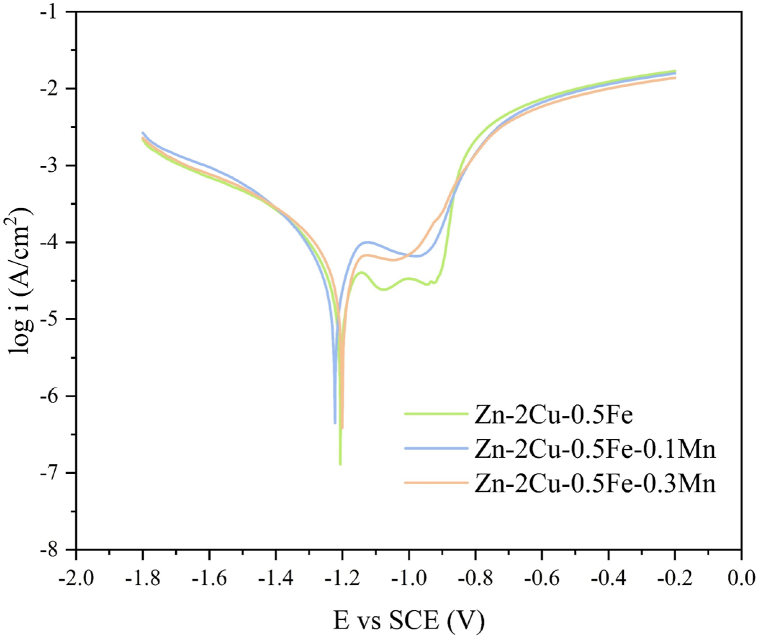
Potentiodynamic polarization curves.

The potentiodynamic polarization curves of the alloys under physiological conditions (in artificial urine at body temperature) are shown in Fig. 5. Furthermore, the corrosion current density (icorr) and corrosion rate (CR) calculated from these curves are presented in Table 3, with the minimum corrosion test duration being no less than 40 min. Among the three alloys, as the Mn content increases, icorr also gradually increases. The icorr values for Zn-2Cu-0.5Fe, Zn-2Cu-0.5Fe-0.1Mn, and Zn-2Cu-0.5Fe-0.3Mn are 15.6 μA/cm^2^, 24.6 μA/cm^2^, and 28.2 μA/cm^2^, respectively, with corresponding CRs of approximately 0.49 mm/year, 0.78 mm/year, and 0.89 mm/year, indicating that the increase in Mn content reduces the corrosion resistance of the alloy. The equation used for the calculation of CR is as follows:

VCR=nicorrFW

where *n* represents the valence of the metal ions, $i_{corr}$ is the corrosion current density, *F* is the Faraday constant, and *W* is the atomic weight of the metal.

**Table 3:** Tafel extrapolation measurements.

Sample	icorr/μA•cm^−2^	CR/mm•y^−1^
S_1_	15.6	0.49
S_2_	24.6	0.78
S_3_	28.2	0.89

Generally, metals with smaller grain sizes exhibit higher corrosion resistance. With the increase in Mn content, the average grain size of the system gradually increases, which is one of the reasons for the increase in corrosion rate with higher Mn content. Additionally, the addition of Mn can enrich the second phase of the alloy; furthermore, the standard electrode potential of Mn is −1.18 V, which is significantly lower than that of Zn (−0.76 V), Cu (0.34 V), and Fe (−0.44 V). Therefore, the incorporation of Mn increases the potential difference between the second phase and the matrix, thereby accelerating the degradation rate [28].

### 3.3 Immersion test result

**Fig. 6:**
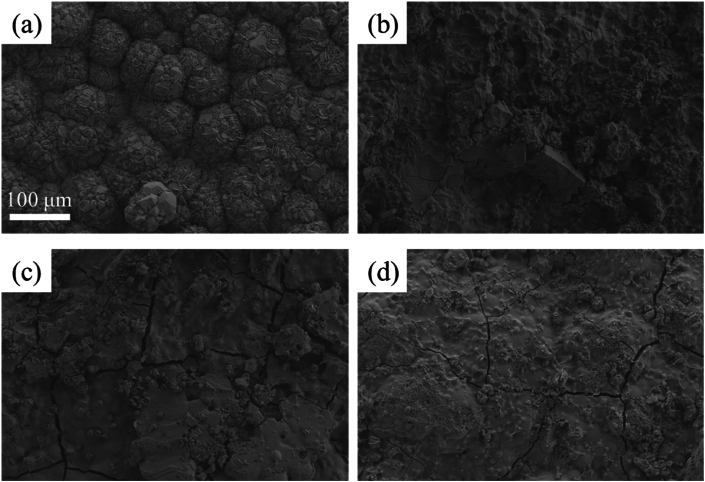
Surface morphology of pure iron (a), Zn-2Cu-0.5Fe (b), Zn-2Cu-0.5Fe-0.1Mn (c), and Zn-2Cu-0.5Fe-0.3Mn (d) cultured in artificial urine for 30 days.

Fig. 6(a–d) shows the surface morphology of pure iron, Zn-2Cu-0.5Fe, Zn-2Cu-0.5Fe-0.1Mn, and Zn-2Cu-0.5Fe-0.3Mn samples immersed in artificial urine for 30 days. It is evident that a large number of calculi form on the surface of pure iron, while fewer calculi form on the surfaces of Zn-2Cu-0.5Fe, Zn-2Cu-0.1Mn, and Zn-2Cu-0.3Mn alloys. This is because pure iron lacks antibacterial properties, making it easy for bacteria to form biofilms on its surface, thereby promoting calculi formation. On the other hand, bacteria accumulate within the calculi, eventually forming bacterial calculi [29]. In contrast, Zn-2Cu-0.5Fe-xMn alloys exhibit strong antibacterial properties. The alloys continuously release Zn and Cu ions through dynamic degradation, making it difficult for bacteria to adhere to and proliferate on their surface, thereby inhibiting the formation of bacterial biofilms and further suppressing calculi formation [29,30].

### 3.4 Antibacterial behavior

**Fig. 7:**
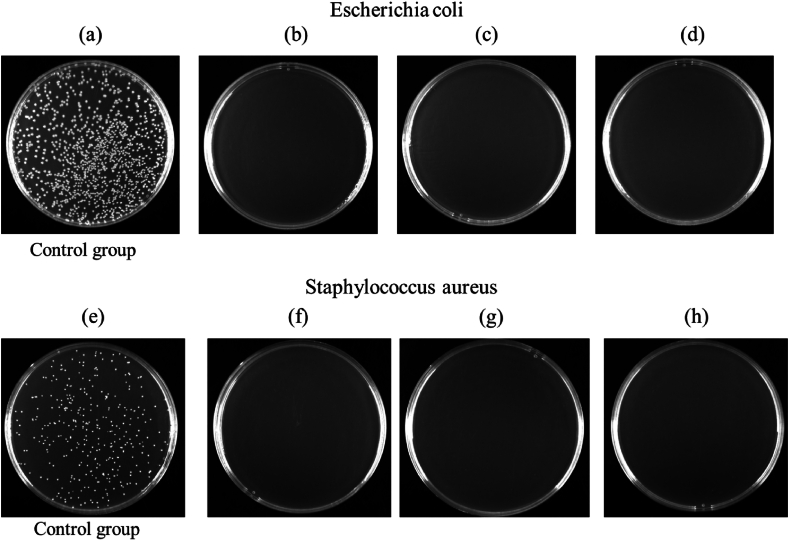
The antibacterial effects of Zn-2Cu-0.5Fe (b, f), Zn-2Cu-0.5Fe-0.1Mn (c, g) and Zn-2Cu-0.5Fe-0.3Mn (d, h) co-cultivated with *Escherichia coli* and *Staphylococcus aureus* for 18 h.

Fig. 7(a–h) illustrates the antibacterial effects of Zn-2Cu-0.5Fe, Zn-2Cu-0.1Mn, and Zn-2Cu-0.3Mn samples after co-culturing with *Escherichia coli* and *Staphylococcus aureus* for 18 h. The antibacterial abilities of the three alloys are strong, with antibacterial rates reaching 100 %. This is because during degradation, all three samples release Zn and Cu ions into the solution. Zn and Cu ions can disrupt the bacterial cell wall and are recognized as highly effective antibacterial agents, which is the main reason for their high antibacterial rates [31,32].

### 3.5 Cytotoxicity

**Fig. 8:**
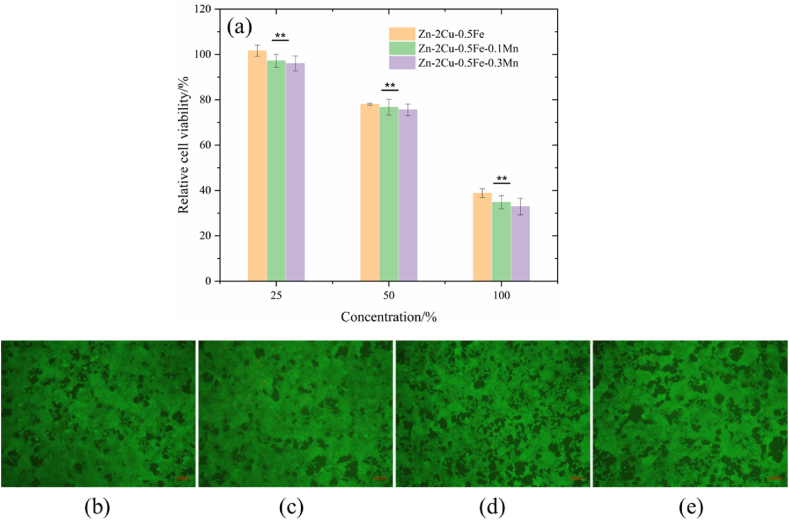
(a) The activity of 293T cells after culture of Zn-2Cu-0.5Fe, Zn-2Cu-0.5Fe-0.1Mn and Zn-2Cu-0.5Fe-0.3Mn at different concentrations for 3 days; (b–e) The fluorescence live/dead staining of control group and 293T cells treated with 25 % concentration of Zn-2Cu-0.5Fe, Zn-2Cu-0.5Fe-0.1Mn and Zn-2Cu-0.5Fe-0.3Mn extracts for 3 days.

Fig. 8 (a) illustrates the viability of 293T cells after a three-day culture period in the presence of extracts at varying concentrations. At a concentration of 100 %, the viability of 293T cells was observed to be less than 50 % for all three alloy extracts. This indicates that at high concentrations, the extracts exhibit significant cytotoxic effects, potentially inhibiting cell growth or inducing cell death. According to ISO 10993–5, the cytotoxicity level is around 3 to 5. When the extracts of the three alloys were diluted to 50 % concentration, the viability of the cells significantly increased (>75 %), indicating a cytotoxicity level of 1. When the extracts of the three alloys were further diluted to 25 % concentration, the viability of the cells was greatly enhanced, reaching close to 100 %. A comparison of cell viability in the extracts of different concentrations of the three alloys clearly shows that with an increase in Mn content, cell viability gradually decreases, indicating a more pronounced toxicity of Mn ions on cells. Therefore, when regulating the composition of the alloy, the Mn content must be controlled within a safe range.

Fig. 8(b–e) show the fluorescence staining of 293T cells after being cultured with 25 % concentration of the extracts of the three alloys for 3 days. At this concentration, all four groups of 293T cells exhibited green skeletons, evenly distributed, and in a healthy state. The cell distribution of the three alloys showed no significant difference from the control group. At a 100 % concentration, the compatibility of 293T cells with the three alloy extracts is poor, primarily due to the high concentration of zinc ions in the extracts. Low concentrations of zinc ions promote cell growth, whereas high concentrations inhibit it [31,32]. When the extracts were diluted to a 50 % concentration, the Zn ion concentration in the two HE sample extracts decreases, making the extracts more conducive to cell growth. Further dilution to 25 % results in a decrease in Zn ion concentration to non-toxic levels, promoting cell proliferation and growth.

## 4 Conclusion

The research investigated the potential of Zn-2Cu-0.5Fe-xMn alloy as a material for ureteral stent. The key findings are as follows.

(1) The average grain size of Zn-2Cu-0.5Fe-xMn and the size of (Fe, Mn)Zn13 phase gradually increased with the increase in Mn content, leading to a decrease in hardness with increasing Mn content.
(2) The corrosion rate of Zn-2Cu-0.5Fe-xMn increased gradually with the increase in Mn content, which was caused by changes in standard electrode potential differences between grain size and elements.
(3) Due to the antibacterial effect of Zn ions and Cu ions, Zn-2Cu-0.5Fe-xMn showed good anti-stone formation ability.
(4) The antibacterial rate of Zn-2Cu-0.5Fe-xMn can reach 100 % with acceptable cytotoxicity.

In summary, Zn-2Cu-0.5Fe-xMn meets the current advanced medical concepts and the essential needs of the human body. By designing the composition, the degradation rate of the material can be controlled, making it a promising important implant material for urology surgery.

## Data availability

Data will be made available on request.

## CRediT authorship contribution statement

**Jiawen Zheng:** Writing – original draft, Methodology, Conceptualization. **Yichun Zheng:** Writing – review & editing, Methodology, Investigation. **Peng Sun:** Visualization, Supervision, Project administration. **Desheng Zhu:** Methodology, Investigation, Conceptualization. **Wentao Fan:** Validation, Methodology, Investigation. **Ting Huang:** Visualization. **Yanfei Fang:** Resources, Conceptualization. **Qing Yang:** Visualization, Resources. **Min Xu:** Writing – review & editing, Methodology, Investigation.

## Declaration of competing interest

The authors declare that they have no known competing financial interests or personal relationships that could have appeared to influence the work reported in this paper.
